# Network pharmacology-based predictions of active components and pharmacological mechanisms of *Artemisia annua* L. for the treatment of the novel Corona virus disease 2019 (COVID-19)

**DOI:** 10.1186/s12906-022-03523-2

**Published:** 2022-03-03

**Authors:** Yexiao Tang, Xiaobo Li, Yueming Yuan, Hongying Zhang, Yuanyuan Zou, Zhiyong Xu, Qin Xu, Jianping Song, Changsheng Deng, Qi Wang

**Affiliations:** 1 Artemisinin Research Center, Guangzhou University of Chinese Medicine, Guangzhou, 510405 China; 2 Sci-tech Industrial Park, Guangzhou University of Chinese Medicine, Guangzhou, 510445 China; 3 Guangzhou Chest Hospital, Guangzhou, 510095 China

**Keywords:** *Artemisia annua* L., COVID-19, Network pharmacology, Molecular docking, Molecule dynamics, MM-GBSA

## Abstract

**Background:**

Novel Corona Virus Disease 2019 (COVID-19) is closely associated with cytokines storms. The Chinese medicinal herb *Artemisia annua* L. (*A. annua*) has been traditionally used to control many inflammatory diseases, such as malaria and rheumatoid arthritis. We performed network analysis and employed molecular docking and network analysis to elucidate active components or targets and the underlying mechanisms of *A. annua* for the treatment of COVID-19.

**Methods:**

Active components of *A. annua* were identified through the TCMSP database according to their oral bioavailability (OB) and drug-likeness (DL). Moreover, target genes associated with COVID-19 were mined from GeneCards, OMIM, and TTD. A compound-target (C-T) network was constructed to predict the relationship of active components with the targets. A Compound-disease-target (C-D-T) network has been built to reveal the direct therapeutic target for COVID-19. Molecular docking, molecular dynamics simulation studies (MD), and MM-GBSA binding free energy calculations were used to the closest molecules and targets between *A. annua* and COVID-19.

**Results:**

In our network, GO, and KEGG analysis indicated that *A. annua* acted in response to COVID-19 by regulating inflammatory response, proliferation, differentiation, and apoptosis. The molecular docking results manifested excellent results to verify the binding capacity between the hub components and hub targets in COVID-19. MD and MM-GBSA data showed quercetin to be the more effective candidate against the virus by target MAPK1, and kaempferol to be the other more effective candidate against the virus by target TP53. We identified *A. annua*’s potentially active compounds and targets associated with them that act against COVID-19.

**Conclusions:**

These findings suggest that *A. annua* may prevent and inhibit the inflammatory processes related to COVID-19.

## Background

Severe acute respiratory syndrome coronavirus 2 (SARS-CoV-2) first appeared in Wuhan, China, in December 2019 and then spread rapidly across China, causing large-scale respiratory infections in the population. So far, we are still unable to stop the transmission of SARS-CoV-2 and the Corona Virus Disease 2019 (COVID-19) continues to threaten human health, causing several deaths daily worldwide. Currently, there are several effective vaccines against SARS-CoV-2, such as mRNA-1273, ChAdOx1 nCoV-19, CoronaVac, and BNT162b2. Although the vaccination program prevented thousands of deaths from COVID-19, it was accompanied by new vaccine-related complications, including vaccine-induced immune thrombocytopenia and thrombosis (VITT) [1], Bell’s palsy [2], and hypersensitivity myocarditis [3]. Therefore, exploration of more effective agents, with a lower incidence of side effects, against COVID-19 is still an urgent requirement. Recently, several novel therapeutic methods have been hypothesized for combating the COVID-19 crisis, including *allium sativum* derived carbon dots, silver nanoparticles, decorin, bilirubin nanomedicine, nanoceria, electric stimulation, and other nanotechnologies, which are justified to be potential theranostic agents for the management of COVID-19 [4–11]. Here, we hypothesized that *Artemisia annua* L. (*A. annua*) could be a promising theranostic agent for COVID-19, so we made many justifications for this hypothesis through data mining.

SARS-CoV-2 can infect the airway cells and result in excessive inflammation leading to a cytokine storm. Pathologically, platelet and endothelial dysfunction are the essential components of COVID-19 infection [12]. Cytokine storm and the elevated levels of circulating cytokines are also associated with various other infectious and immune-mediated conditions as seen in COVID-19 [13].

*A. annua*, which is known to possess antiviral abilities [14, 15], is suggested by Haq et al. [16] to be of clinical importance in this epidemic and make a case for more elaborated clinical trials and experimental studies to explore its effects on the SARS-CoV-2 virus. Furthermore, some components of *A. annua* have been reported to have effective antiviral properties and immunosuppressive effects in vivo [17]. Artemisinin, an antimalarial lactone derived from *A. annua,* is reported to decrease the infiltration of immunomodulatory cells and inflammatory cytokines in vivo [18]. The use of Artemisinin against different respiratory diseases has also been investigated in lung cancer models and inflammatory-driven respiratory disorders [19]. Isohamnetin, an active component of *A. annua,* an in vivo study showed it can inhibit SARS-CoV-2 spike pseudotyped virus entering ACE2h cells [20]. Quercetin, belonging to the active component of *A. annua*, a comprehensive review summarized it seems to protect against SARS-CoV-2 through different mechanisms of action [21]. A previous stiduy indicated that another active component of *A. annua* named kaempferol may potentially interact with the SARS-CoV-2 main protease 3CLpro [22]. These studies above demonstrated the anti-SARS-CoV-2 activity of *A. annua*.

Given these findings, there is still a lack of direct evidence proving that *A. annua* may play an important role in COVID-19 management. Therefore, it is essential to investigate the potential active components of *A. annua* against COVID-19 and figure out their mechanisms of action. Network pharmacology, which holds a systematic and holistic view in understanding the nature of drugs, has been endorsed by various British pharmacologists as a mean to represent datasets and reveal the nature of the interactions between several nodes [23, 24]. These nodes represent small molecules like genes and proteins.

**Fig. 1:**
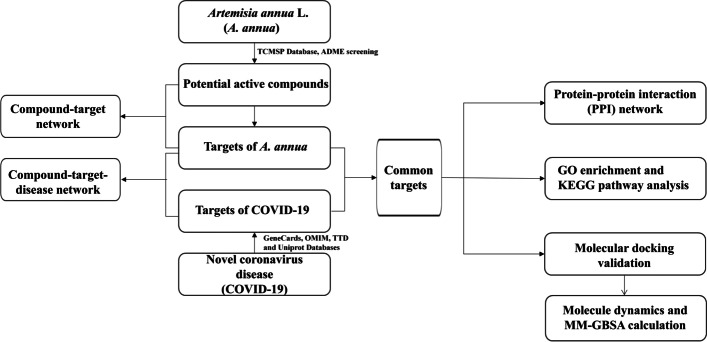
The flowchart of this study

Our study aims at exploring the relevant roles of the potentially active components of *A. annua* for the treatment of COVID-19 using network pharmacology tools. Through the complex network analysis of drug-target-disease signaling pathway, we lay the foundation for further study on *A. annua* and *A.annua*-based drugs to treat COVID-19, and provide a platform to show the effectiveness of Chinese medicine against this epidemic. The process of this research is shown in Fig. 1.

## Methods

### Active compounds screening

The Traditional Chinese Medicines for Systems Pharmacology Database and Analysis Platform (TCMSP, available online: https://www.tcmsp-e.com), a pharmacology platform built for herbal medicines, was used to gather all the chemical components of *A. annua* [25]. Potential active components were defined as ingredients with OB ≥ 30% and DL ≥ 0.18. We further identified effective targets of each compound by DrugBank Database (https://www.drugbank.ca) [26].

### Compound-target network construction

Same targets generated by the same-compound prediction were screened and removed to avoid repetition and were then entered into UniProt Database (https://www.uniprot.org) [27]. The search for the gene symbols of the active compounds in *A. annua* was refined by selecting species as ‘*Homo sapiens*’, so as to exclude non-human, irregular, and repetitive targets. Compounds and targets data were finally introduced into Cytoscape 3.7.2 software to build a compound-target (C-T) network.

### COVID-19 related targets collection

COVID-19 related targets were obtained by GeneCards Database (https://www.genecards.org) [28]. We searched for the keywords Novel Coronavirus Pneumonia, COVID-19, SARS-COV-2, and calculated the correlation between the targets and COVID-19 through the Gifts algorithm. Targets with a score of 30 were selected as the potential COVID-19 targets according to their correlation ranking and the standard gene names of the targets were also figured out. Additionally, OMIM (https://omim.org) [29] and TTD databases (http://db.idrblab.net/ttd) [30] were used to obtain more targets.

### Compound-target-disease network and protein-protein interaction (PPI) construction

The compound-target-disease (C-T-D) network was constructed on the direct therapeutic targets of *A. annua* that acts on COVID-19. The common intersection for *A. annua* and COVID-19 was obtained by Venny online analysis tool [31], and the selected common targets were uploaded to String 11.0 Database (https://string-db.org, version 11.0) to construct the PPI network. The organismic selection was for ‘*Homo sapiens*’, and the minimum required interaction score was set to ‘medium confidence (>0.400)’. The topological importance of nodes in the C-T-D network was evaluated by the degree, betweenness centrality, and closeness centrality [32]. A node was considered a hub when the degree of the node was more than the twofold average degree value [33]. Subsequently, three topological parameters were computed to pick up big hubs, in which the degree, betweenness centrality, and closeness centrality were greater than the median value of all hubs in the network [34].

### Gene ontology (GO) enrichment analysis

GO functional enrichment analysis, such as biological process (BP), cellular component (CC) and molecular function (MF) of target proteins of *A. annua* acting on COVID-19, was performed using David 6.8.0 Database (https://david.ncifcrf.gov) [35]. An adjusted *P*-value of 0.05 was considered to identify the enriched terms.

### Kyoto encyclopedia of genes and genomes (KEGG) pathway enrichment analysis

KEGG Database (https://www.kegg.jp/kegg/) [36–38] was used to perform KEGG pathway enrichment analysis of target proteins of *A. annua* acting on COVID-19. KEGG Mapper queried the location of common targets between A.annua and SARS-CoV-2 and shared targets in the key pathway.

### iGemdock molecular docking

The structures of the hub targets were downloaded from PDB (http://www.pdb.org) [39] and saved as PDB files, and the structures of the hub compounds were obtained from TCMSP and saved as mol2 files. Before docking, polar hydrogens were added to target proteins, and water molecules and original ligands were removed from the target proteins using Discovery Studio (Version 2016). Then, all the removed ligands were also saved as mol2 files for further docking. Further, iGEMDOCK (http://gemdock.life.nctu.edu.tw/dock, Version 2.1) [40] was used to complete Molecular docking simulation. The default parameters were set to ‘standard docking’ and ‘docking accuracy settings’. In general, the binding affinity is determined by the binding free energy, which is relatively low when the conformation of the binding is stable.

### GlideXP molecular docking

The composite system of Isorhamnetin, Quercetin, Kaempferol, and each protein using XGlide module in the Schrödinger2018–1 software. The 3D structure of the small molecule was downloaded from Pubchem. For the protein structure, we downloaded it from the PDB database. The PDB IDs were 3 W55 (MAP1), 5 T89 (VEGFA), 6MXY (TP53), 6SFJ (MAP8), 6ZR5 (MAP14). Subsequently, the Protein Preparation Wizard in Schrödinger was used to optimize each protein separately, including removing non-ligand molecules and water molecules, adding hydrogen atoms, and using the OPLS2005 force field to optimize the structure remove intermolecular collisions. We finally docked the prepared files through the XGlide docking module, used a sitemap to predict the docking site, and the standard precision (SP) algorithm for molecular docking.

### Molecular dynamics simulations

The small molecule-protein complex system obtained by docking was used as the initial structure to perform all-atom molecular dynamics simulations. The charge of the small molecule was calculated by the antechamber module and the Hartree–Fock (HF) SCF/6-31G* of the Gaussian 09 software [41, 42]. Small molecules and proteins were described using GAFF2 small molecule force field and ff14SB protein force field, respectively [43, 44]. The four systems all used the LEAP module to add hydrogen atoms to the system, add a truncated octahedral TIP3P solvent box five at a distance of 8 Å from the system [45], and add Na^+^/Cl^−^ to the system to balance the system charge.

The molecular dynamics simulation was performed with AMBER 18 software [46]. Before the simulation, energy optimization was performed on the system, including the steepest descent method with 2500 steps and the conjugate gradient method with 2500 steps. After the system energy optimization was completed, the system’s temperature was increased by 200 ps under a fixed volume and a constant heating rate so that the temperature of the system slowly rose from 0 K to 298.15 K. Under the condition that the system maintains a temperature of 298.15 K, a 500 ps NVT (isothermal and isopyknic) system simulation was performed to distribute the solvent molecules in the solvent box uniformly. Then, in the case of NPT (isothermal and isobaric), a 500 ps equilibrium simulation was performed on the entire system. Finally, under periodic boundary conditions, the four composite systems were subjected to 4 ns NPT ((isothermal and isobaric) system simulations. In the simulation, the cutoff distance of the non-bond was set to 10 Å, the Particle mesh Ewald (PME) method was used to calculate the long-range electrostatic interaction [47], the SHAKE method was used to limit the length of the hydrogen atom bond [48], and the Langevin algorithm was used for temperature control [49], the collision frequency γ was set to 2 ps^− 1^. The system pressure was 1 atm, the integration step was 2 fs, and the trajectory was saved every 4 ps for subsequent stability analysis and binding free energy calculation.

### Binding free energy calculations

We calculated the free energy of binding between proteins and ligands in all systems using the MM-GBSA method [50–53]. In this study, the MD trajectory of 2–4 ns was used for calculation, and the specific formula is as follows:

1 $${\Delta G}_{bind}={\Delta \mathrm{G}}_{\mathrm{complex}}-\left({\Delta \mathrm{G}}_{\mathrm{receptor}}+{\Delta \mathrm{G}}_{\mathrm{ligand}}\right)={\Delta \mathrm{E}}_{\mathrm{internal}}+{\Delta \mathrm{E}}_{\mathrm{VDW}}+{\Delta \mathrm{E}}_{\mathrm{elec}}+{\Delta \mathrm{G}}_{\mathrm{GB}}+{\Delta \mathrm{G}}_{\mathrm{SA}}$$

In formula (1), ΔE_internal,_ ΔE_VDW,_ and ΔE_elec_ represents internal energy, van der Waals interaction, and electrostatic interaction, respectively. The internal energy includes bond energy (E_bond_), angular energy (E_angle_), and torsion energy (E_torsion_), and ΔG_GB_ and ΔG_SA_ collectively referred to as solvation free energy (ΔG_GB_ is the free energy of polar solvation, and ΔG_SA_ is the free energy of non-polar solvation). We used the GB model (*igb* = 2) developed by researchers such as Nguyen [54] to calculate ΔG_GB_. ΔG_SA_ was calculated based on the product of surface tension (γ) and solvent accessible surface area (SA), ΔG_SA_ =0.0072 × ΔSASA [55]. We ignored Entropy changes due to high computational resources and low precision [50, 51].

## Results

### Active compounds selection

19 active compounds found in *A. annua* were screened by TCMSP platform under the standard conditions for a drug, particularly OB ≥ 30% and DL ≥ 0.18, including eupatin, isorhamnetin, sitosterol, etc. The OB and DL values for each ingredient are shown in Table 1.

**Table 1:** Potential active compounds and ADME parameters of *A. annua*

Name	Mol ID	Molecule Name	OB (%)	DL
*A. annua*1	MOL002235	eupatin	50.8	0.41
*A. annua*2	MOL000354	isorhamnetin	49.6	0.31
*A. annua*3	MOL000359	sitosterol	36.91	0.75
*A. annua*4	MOL004083	tamarixetin	32.86	0.31
*A. annua*5	MOL004112	patuletin	53.11	0.34
*A. annua*6	MOL000422	kaempferol	41.88	0.24
*A. annua*7	MOL000449	stigmasterol	43.83	0.76
*A. annua*8	MOL004609	areapillin	48.96	0.41
*A. annua*9	MOL005229	artemetin	49.55	0.48
*A. annua*10	MOL000006	luteolin	36.16	0.25
*A. annua*11	MOL007274	skrofulein	30.35	0.3
*A. annua*12	MOL007401	cirsiliol	43.46	0.34
*A. annua*13	MOL007404	vitexin_qt	52.18	0.21
*A. annua*14	MOL007412	DMQT	42.6	0.37
*A. annua*15	MOL007415	[(2S)-2-[[(2S)-2-(benzoylamino) -3-phenylpropanoyl]amino]-3-phenylpropyl] acetate	58.02	0.52
*A. annua*16	MOL007423	6,8-di-c-glucosylapigenin_qt	59.85	0.21
*A. annua*17	MOL007424	artemisinin	49.88	0.31
*A. annua*18	MOL007426	deoxyartemisinin	54.47	0.26
*A. annua*19	MOL000098	quercetin	46.43	0.28

### Compound-target network and compound-target-disease network

**Fig. 2:**
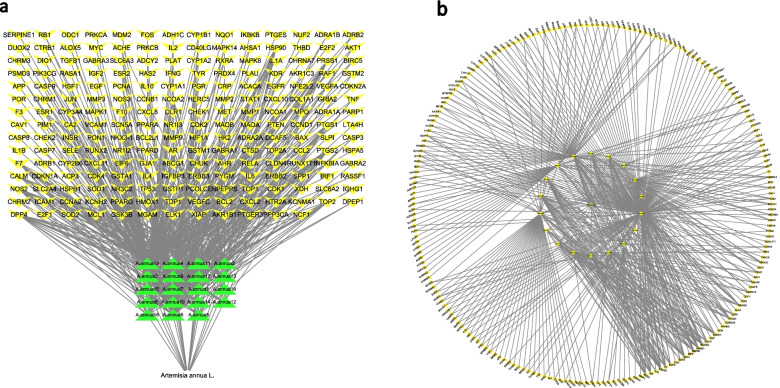
Compound-target network of *A. annua* (**a**) and Compound-target-disease network (**b**)

We got 19 compound nodes and 208 target nodes (Fig. 2a). We obtained 871 COVID-19 targets by merging and deleting the duplicate values. From the 871 COVID-19 target genes retrieved, the C-T-D network comprised of 88 nodes and 229 edges. The details are presented in Fig. 2b.

### Protein-protein interaction (PPI) construction

**Fig. 3:**
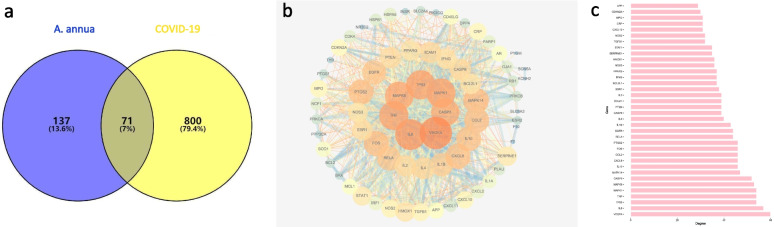
Protein-protein interaction (PPI) of *A. annua* and COVID-19 (**a**, **b**) and degree values of critical nodes in the PPI network (**c**). Visualization parameters of the network: (1) Map node size to degree: low values to small sizes. (2) Map node color to degree: low values to dark colors. (3) Map edge size to combined- score: low values to small sizes. (4) Map edge color to combined- score: low values to bright colors

The screened active compounds of *A. annua* were intersected with the COVID-19 related targets, and the Venn analysis tool was used to obtain 71 common targets (Fig. 3a). The PPI network diagram contained 71 nodes and 1071 edges (Fig. 3b). 36 key nodes were selected as hubs according to the used standard (degree value > mean value) with the cutoff of twofold median value of degree (degree> 28.65). The nodes obtained were VEGFA, IL6, TP53, TNF, MAPK1, MAPK8, CASP3, MAPK14, IL10, CXCL8, CCL2, FOS, PTGS2, RELA, EGFR, IL1B, IL4, CASP8, PTEN, ICAM1, IL2, ESR1, BCL2L1, IFNG, PPARG, NOS3, HMOX1, SERPINE1, STAT1, TGFB1, NOS2, CXCL10, CRP, MPO, CDKN2A and AP. VEGFA was the node with the highest degree value as 60 (Fig. 3c). With a value of 57, IL6 stood as the second highest node. TP53, TNF, and MAPK1 had the same degree value of 54, IL10 had a value of 46, IL4 a value of 40, and IL2 a value of 39. The higher the node value of the target, the more important role it played in the network regulation and hence was likely to be a key target for *A. annua* to treat COVID-19.

### GO enrichment analysis

**Fig. 4:**
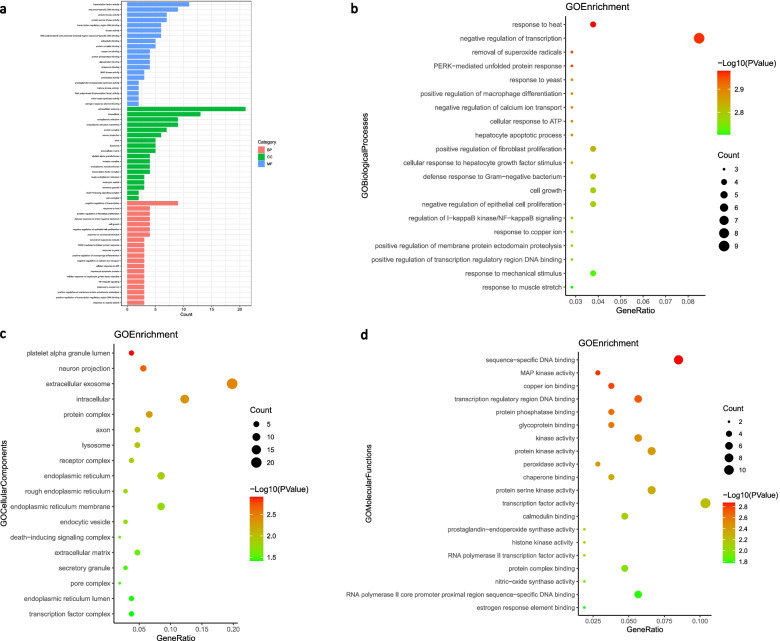
GO enrichment analysis. Histogram of GO enrichment analysis (**a**), scatter plots of mainly Gene Ontology terms for biological process (**b**), cellular component (**c**) and molecular function (**d**)

GO function enrichment analysis was carried out with the help of DAVID database. 545 GO entries were obtained and 429 biological processes (BP). Mainly a negative regulation of transcription was observed. Other observations were related to response to heat, positive regulation of fibroblast proliferation, defense response to Gram-negative bacterium, cell growth, negative regulation of epithelial cell proliferation, response to mechanical stimulus, removal of superoxide radicals, PERK-mediated unfolded protein response, and a positive regulation of macrophage differentiation. GO analysis also highlighted 44 cellular components (CC), mainly extracellular exosome, intracellular, endoplasmic reticulum, endoplasmic reticulum membrane, protein complex, neuron projection, axon, lysosome, extracellular matrix, and platelet alpha granule lumen. 72 molecular functions (MF), mainly transcription factor activity, sequence-specific DNA binding, protein kinase activity, protein serine kinase activity, transcription regulatory region DNA binding, kinase activity, RNA polymerase II core promoter proximal region sequence-specific DNA binding, calmodulin binding, and protein complex binding, were seen. 58 GO entries were finally screened out, as shown in Fig. 4.

### KEGG pathway enrichment analysis

**Fig. 5:**
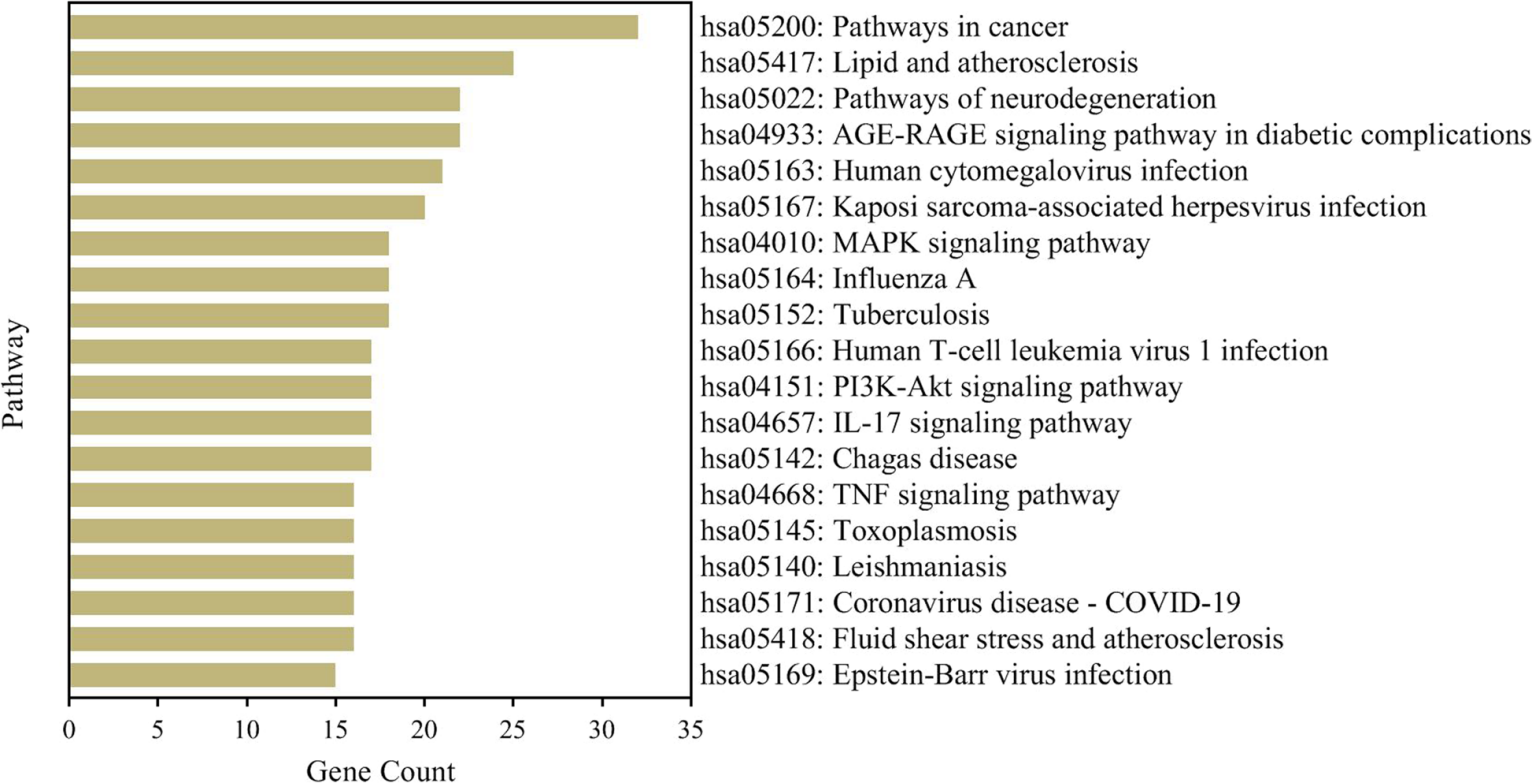
KEGG pathway enrichment analysis

**Fig. 6:**
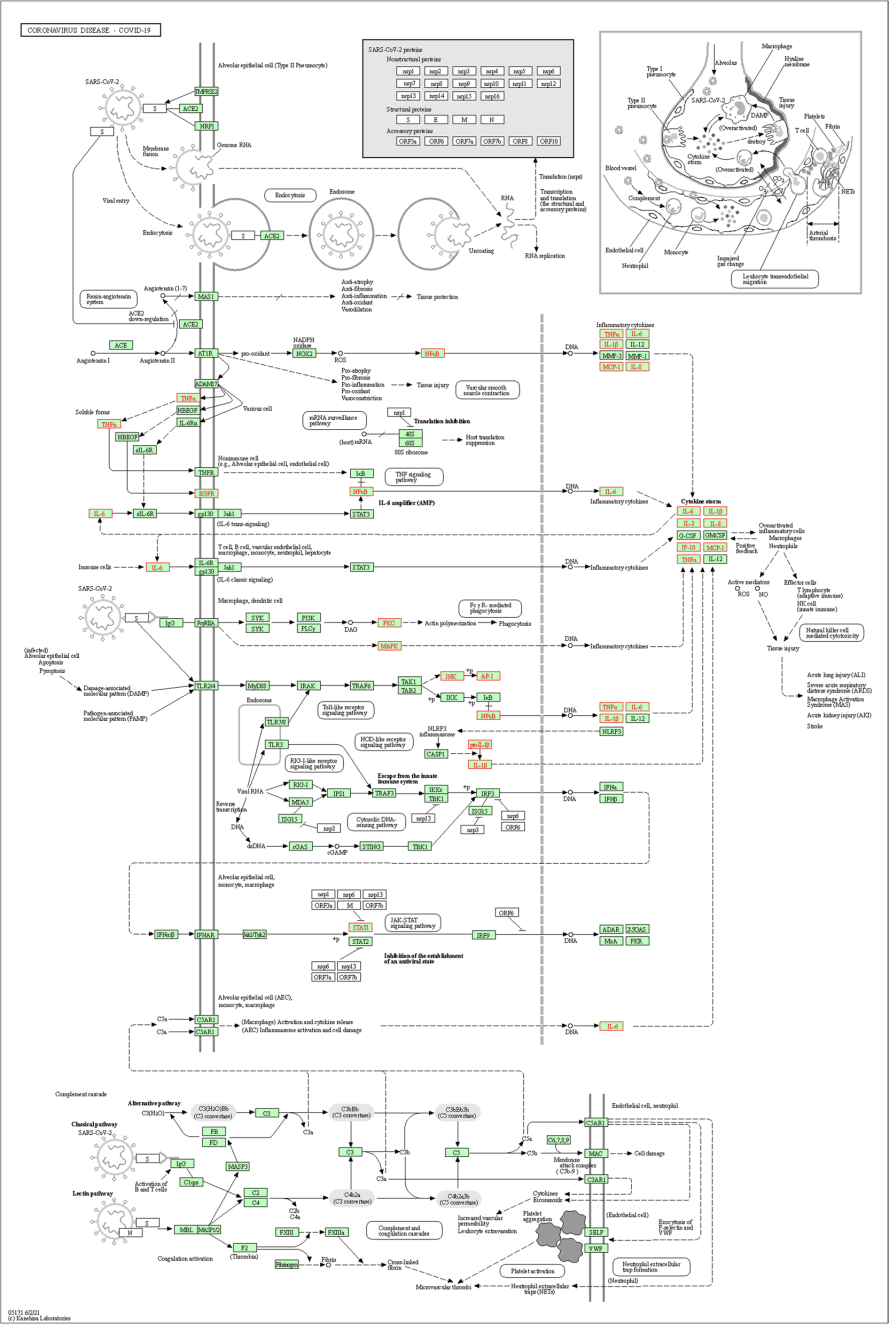
Roles in the reaction related to *A. annua* in anti- SARS-CoV-2. The red nodes represent represents overlapping targets between *A. annua* and SARS-CoV-2

To clarify the role of *A. annua* targets in the signaling pathway, analysis of KEGG pathway enrichment was performed using the KEGG database. The analysis results showed 221 pathways of *A. annua* related to the COVID-19. The first 20 KEGG pathways were finally screened out by gene count and shown in Fig. 5. Then the COVID-19 pathway and the location of SARS-CoV-2 targets and overlapping genes of enriched pathways are listed in Fig. 6.

### iGemdock molecular docking analysis

The hubs namely quercetin, isorhamnetin and kaempferol, with above-average values for degree (> 28.65), betweenness (> 0.0128) and closeness (> 0.619), were simultaneously regarded as the hub compounds. The details are presented in Table 2.

**Table 2:** Details of Selected Hub Targets

Targets	Betweenness Centrality	Closeness Centrality	Degree
VEGFA	0.05006389	0.875	60
MAPK1	0.03798558	0.81395349	54
TNF	0.02542764	0.81395349	54
TP53	0.03216216	0.79545455	54
MAPK8	0.02504844	0.8045977	53
CASP3	0.02525257	0.79545455	52
MAPK14	0.01840782	0.74468085	47

**Fig. 7:**
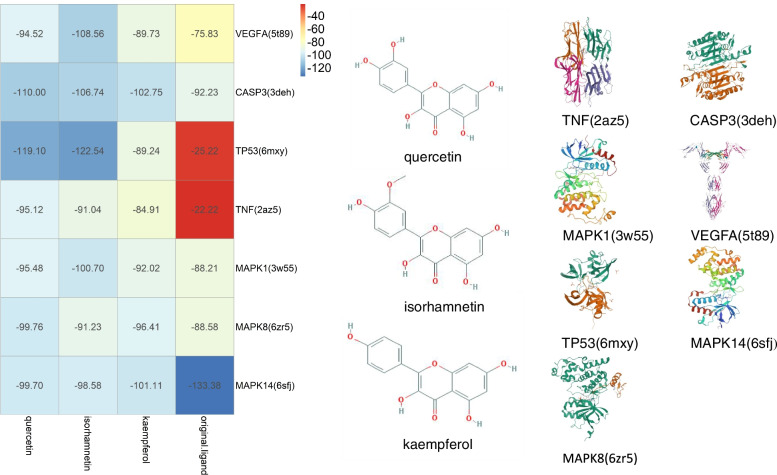
Structures of hub compounds and targets of *A. annua* for COVID-19 and Heatmap of Binding Energy by iGemdock molecular docking Analysis

We considered the median value of all pairs of binding energy as a strong binding efficacy of less than − 99.49 KJ/mol. The docking simulation results indicated that isorhamnetin and quercetin exhibited excellent binding affinity, and the details on the binding energy are presented in Fig. 7. The binding energy between the removed ligands and their target proteins was calculated in order to further investigate the binding affinity. Our results showed that the binding affinity of isorhamnetin-TP53 and quercetin-TP53 were far more superior than that of the removed original ligands and targets. Thus, these results indicated that isorhamnetin and quercetin were the potential therapeutic active compounds and TP53 was the potential therapetic target.

### GlideXP molecular docking analysis

**Fig. 8:**
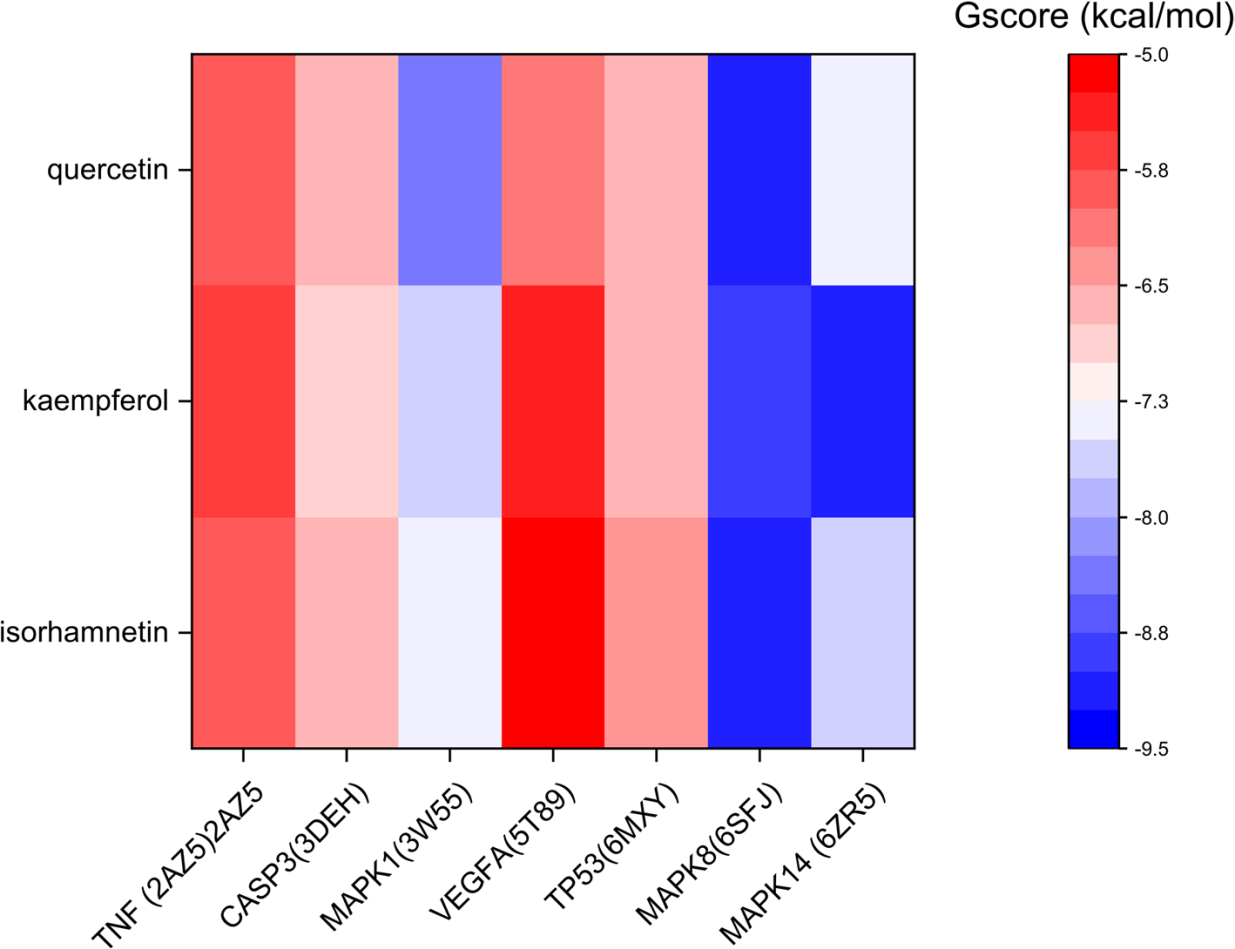
Heatmap of Gscore by GlideXP molecular docking analysis. The color from red to blue indicates that the binding ability is weak to strong

To further enhance the study’s credibility, we used the GlideXP high-precision docking algorithm to obtain the combination of the above seven hub targets and the three hub active components. The active site parameters of each target were calculated and are listed in Table 3. The lower docking affinity reflects the stronger binding ability between components and targets. As shown in Table 3 and Fig. 8, The combination with a value lower than − 8.00 kcal/mol binds more tightly, suggesting that MPK1, MPK8, and MPK14 are essential potential targets. Therefore, *A.annua* may improve COVID-19 by regulating the activity of these proteins. Then we used molecular dynamics simulation to study more in-depth.

**Table 3:** The docking scores of different combinations of complexes

Target	Ligand	GScore
TNF(2AZ5)	isorhamnetin	− 5.90
TNF(2AZ5)	quercetin	−5.76
TNF(2AZ5)	kaempferol	−5.62
CASP3(3DEH)	kaempferol	−6.91
CASP3(3DEH)	quercetin	−6.68
CASP3(3DEH)	isorhamnetin	−6.61
MAPK1(3 W55)	quercetin	−8.33
MAPK1(3 W55)	kaempferol	−7.62
MAPK1(3 W55)	isorhamnetin	−7.48
VEGFA(5 T89)	quercetin	−6.07
VEGFA(5 T89)	kaempferol	−5.41
VEGFA(5 T89)	isorhamnetin	−5.15
TP53(6MXY)	quercetin	−6.64
TP53(6MXY)	kaempferol	−6.57
TP53(6MXY)	isorhamnetin	−6.29
MAPK8(6SFJ)	isorhamnetin	−9.08
MAPK8(6SFJ)	quercetin	−9.07
MAPK8(6SFJ)	kaempferol	−8.83
MAPK14(6ZR5)	kaempferol	−9.10
MAPK14(6ZR5)	isorhamnetin	−7.58
MAPK14(6ZR5)	quercetin	−7.39

### MD simulation analysis

**Fig. 9:**
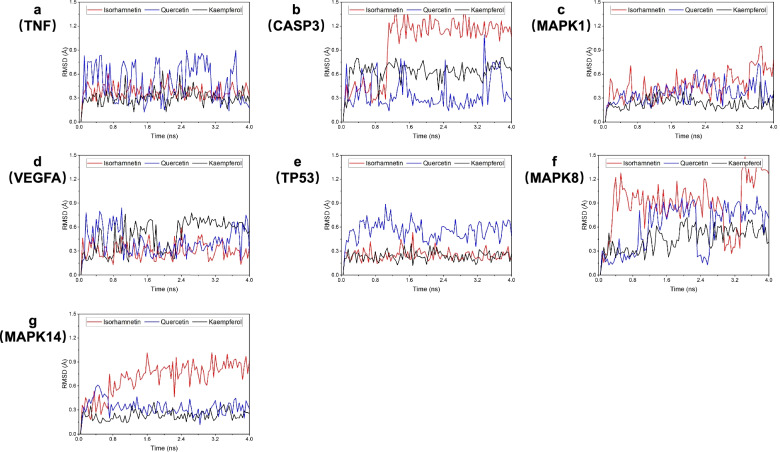
Analysis of molecular dynamics simulation results. **a** Root mean square deviation (RMSD) of backbone atoms of TNF in complex with isorhamnetin, quercetin and kaempferol, **b** RMSD of backbone atoms of CASP3 in complex with isorhamnetin, quercetin and kaempferol, **c** RMSD of backbone atoms of MAPK1 in complex with isorhamnetin, quercetin and kaempferol, **d** RMSD of backbone atoms of VEGFA in complex with isorhamnetin, quercetin and kaempferol, **e** RMSD of backbone atoms of TP53 in complex with isorhamnetin, quercetin and kaempferol, **f** RMSD of backbone atoms of MAPK8 in complex with isorhamnetin, quercetin and kaempferol, **g** RMSD of backbone atoms of MAPK14 in complex with isorhamnetin, quercetin and kaempferol

Based on the binding modes of three natural products and four potential targets, we tried to further explore the stability of these 21 complexes through molecular dynamics simulation technology to clarify which complexes are more reliable. The results are shown in Fig. 9, which shows the root mean square fluctuations (RMSD) of 21 kinds of compounds in the MD process and the stability of the compound with the fluctuations. In contrast, most of the stablest fluctuations are the complex system composed of MAPK1, consistent with those mentioned above GlideXP molecular docking results. Figure 9 shows the fluctuation of the ligand during the complex dynamic simulation. The more violent the fluctuation, the more unstable it is. During dynamic simulation, the RMSD of each molecule in the corresponding protein changed with the simulation time to show the stability of the numerical reaction molecule at the active site. The lower the fluctuation, the more stable the binding.

### MM-GBSA calculations

In addition to investigating the stability between the three compounds and the four targets, we also adopted the high-precision binding free energy calculation method MM-GBSA to study the binding free energy of these complex systems more directly.

**Fig. 10:**
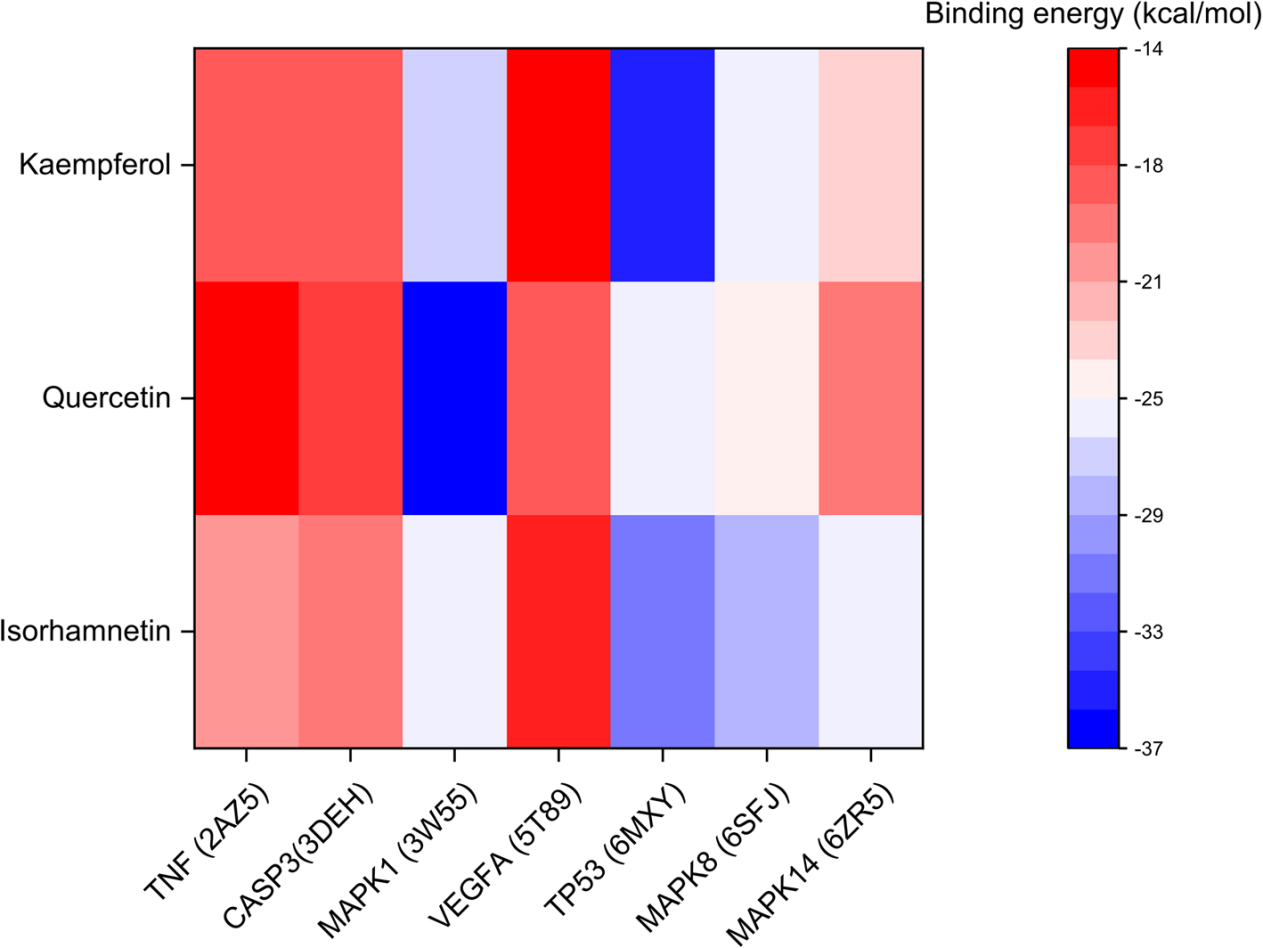
Binding free energy heatmap based on MM-GBSA calculation. The color from red to blue indicates that the binding free energy is weak to strong

Table 4 and Fig. 10 show the specific results. We obtained the binding free energy of 21 systems through MM-GBSA calculation and decomposed the energy term. Consistent with the above calculations, the highest binding free energy combination was the hybrid system of MAPK1/Quercetin, and its binding free energy was − 36.7 kcal/mol. The higher combination was TP53/Kaempferol, and its binding free energy was − 34.33 kcal/mol. Our analysis showed that these high binding free energy combinations were mainly contributed by van der Waals forces and electrostatic interactions. In summary, we believe that the MAPK1/Quercetin, TP53/Kaempferol complex has a high degree of credibility and is necessary for further experimental verification.

**Table 4:** Binding Free Energy (kcal/mol) based on MM-GBSA calculations

Target	Isorhamnetin	Quercetin	Kaempferol
TNF (2AZ5)	−20.29	−14.65	−18.76
CASP3(3DEH)	−20.08	−17.4	−18.18
MAP1 (3 W55)	−26.25	−36.7	−27.23
VEGFA (5 T89)	−16.03	−18.17	−13.78
TP53 (6MXY)	−31.39	−25.64	−34.33
MAP8 (6SFJ)	−28.01	−24.74	−25.64
MAP14 (6ZR5)	−25.48	−20.01	−23.73

### Binding mode analysis

**Fig. 11:**
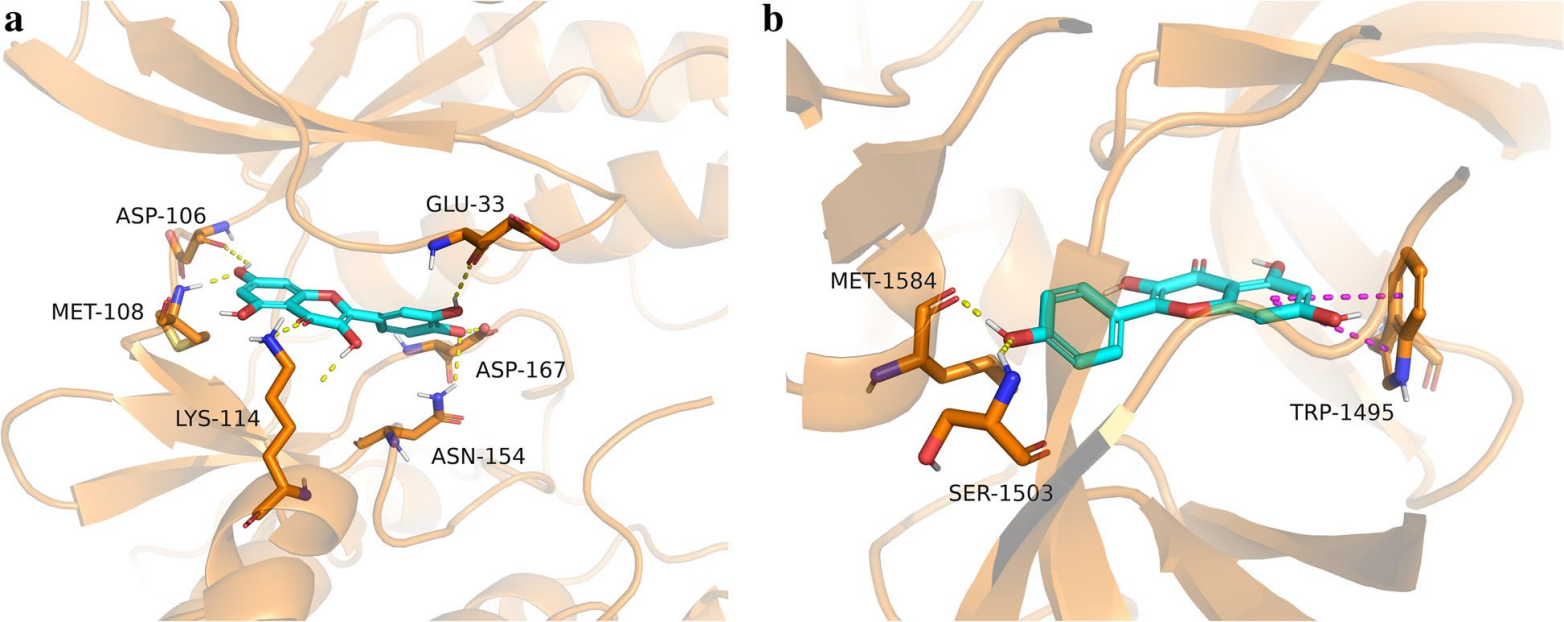
The binding mode analysis. The yellow dashed line indicates the hydrogen bonding effect, and the wine red dashed line indicates the pipi-stack effect. **a** The binding mode of MAPK1/Quercetin complex, **b** TP53/Kaempferol complex

Complexes such as MAPK8/Isorhamnetin, MAPK8/Quercetin, MAPK8/Kaempferol, and MAPK14/Kaempferol were good interactions during GlideXP molecular docking binding free energy. They all displayed negative binding free energy values excluded from further analysis. MAPK1/Quercetin has good interaction during GlideXP molecular docking and favorable binding free energy. Although Complex TP53/Kaempferol did not display good interaction during GlideXP molecular docking, it was found to have good interaction during iGemdockimolecular docking and showed good binding free energy values. Therefore, we selected MAPK1/Quercetin and TP53/Kaempferol complexes to verify their binding mode. Figure 11 shows that the binding mode of MAPK1/Quercetin and TP53/Kaempferol complexes combine well. The formation of hydrogen bonds between quercetin and the active site residues of MAPK1 involved residues ASP106, MET108, LYS114, ASN154, and ASP167 (Fig. 11a). The formation of hydrogen bonds between kaempferol and the active site residues of TP53 involved residues MET1584 and SER1503 (Fig. 11b). Hence, our findings confirmed that *A.annua* might treat COVID-19 by regulating the activity of MAPK1 and TP53.

## Discussion

Besides *A. annua*’s wide usage as an antimalarial drug, there are many clinical and experimental studies showing that *A. annua* also has antiviral and anti-inflammatory properties. Mathieu Gendrot et al. conducted an inhibition test of SARS-CoV-2 replication in Vero E6 cells with 5 artemisinin-based combination therapies (ACTs) in vitro. In their study, mefloquine-artesunate exerted the highest antiviral activity with 72.1–18.3% inhibition at the expected maximum blood concentration (C_max_) for each ACT drug, at doses commonly administered in malaria treatment. Additionally, all the other combinations include artesunate-amodiaquine, artemether-lumefantrine, artesunate-pyronaridine, or dihydroartemisinin-piperaquine, showed antiviral inhibition in the same ranges (27.1 to 34.1%) [56]. Therefore, we speculated that *A. annua* may have a significant effect on COVID-19 as well. In our study, we elucidated the relationship between *A. annua* and COVID-19 using a network pharmacology strategy. We screened 19 potential active ingredients and 208 potential targets of *A. annua* and obtained 71 common targets for *A. annua* and COVID-19. We constructed networks such as compound-target network, PPI network of *A. annua* and COVID-19, and performed GO and KEGG enrichment analysis to comprehensively understand the mechanisms behind *A. annua*’s capabilities in treating COVID-19.

In addition to the overall efficacy in clearing heat and detoxifying properties, each compound of *A. annua* can also play different roles. As shown by the C-T network analysis, quercetin, isorhamnetin and kaempferol compounds play a crucial role in manifesting treatment properties of *A. annua* in treating COVID-19. Quercetin can also prevent the virus from sticking to cells by interacting with proteins on the surface of the virus [57] and by inhibiting inflammatory cytokines [58]. Isorhamnetin owns strong antiviral potency, which can block cytoplasmic lysosome acidification and reduce virus-induced ROS generation [59]. Kaempferol is known to significantly suppress cell-autonomous immunity through down-regulation of p38 and JNK [60]. Interestingly, its derivatives are also considered to have antiviral properties against the 3a channel protein of coronavirus, a channel that is related to the viral release mechanism [61]. Artemisinin and its derivations were beneficial for the management of viral infection, and the antiviral effect is associated with enhanced type I interferon response of the host [62, 63]. Thus Quercetin, isorhamnetin, kaempferol, and artemisinin could be the main potentially active compounds of *A. annua* that are employed in the management of COVID-19 by inhibiting inflammatory mediators.

PPI network analysis showed that the degrees and betweenness centrality of 36 key genes were greater than the average. The further topology analysis of the C-T-D network revealed that 7 key target proteins include key genes for the pathological mechanisms of COVID-19, mainly the immune and the blood coagulation mechanism. For instance, vascular endothelial growth factor-A (VEGF-A) is a crucial regulator of angiogenesis, a process that forms new blood vessels from pre-existing vessels [64, 65]. Clinical data showed that several COVID-19 patients are presented with increasing levels of angiogenesis and endotheliopathy markers [66, 67]. Additionally, brain inflammation caused by SARS-CoV-2 was considered to be related to VEGFA, which can facilitate the accumulation of inflammatory cells and regulate the angiopoietins II level [68]. TNF is an intensely studied proinflammatory cytokine, which can trigger either cell survival or cell death by regulating a complex inflammatory network [69, 70], and its hyperproduction is related to the pathogenesis of COVID-19 as well. The caspase family regulates crucial biological functions, such as cell death in apoptosis and pyroptosis, as well as performs non-cell death functions in inflammation, dendrite trimming, and cell differentiation [71]. A spatially restricted activation of caspase-3 occurs in maturing megakaryocytes to promote proplatelet maturation and platelet shedding in the bloodstream [72].

TP53 is a transcription factor that functions towards the regulation of important cellular activities, such as cell cycle, senescence, and apoptosis, which can suppress inflammation in a plethora of human tissues [73] and thus is often mutated in certain malignancies. The increased MAPK activation may influence the release of many proinflammatory cytokines [74] and induce platelet aggregation in COVID-19 patients [75]. Based on the above details, we find that *A. annua* can play a therapeutic role in the entire process of COVID-19 management thus, meeting the need for the systematic treatment of COVID-19 and futuristic studies, it would be worthwhile to explore the role of VEGFA, CASP3, TP53, TNF, MAPK1, MAPK8 and MAPK14 in the mechanisms manifested by *A. annua*. Through molecular docking simulation and computing binding free energy, we validated that 3 hub compounds quercetin, isorhamnetin, and kaempferol, particularly quercetin and isorhamnetin, had an excellent binding affinity with the 7 hub targets. The MD and MM-GBSA calculation data further indicated that MPK1/Quercetin and TP53/Kaempferol possessed the highest binding free energy, which demonstrated the repurposing possibilities of quercetin and kaempferol based on their binding activity with multiple COVID-19 targets, and supported their ability to function as anti-SARS-CoV-2.

The GO enrichment analysis revealed that the treatment of COVID-19 with *A. annua* was mainly related to BP, CC, and MF. BP includes negative regulation of transcription, response to heat, and positive regulation of fibroblast proliferation. CC includes extracellular exosome, intracellular, and endoplasmic reticulum. MF includes transcription factor activity, sequence-specific DNA binding, and protein kinase activity. The KEGG pathways of *A. annua* are mainly related to the roles of immune regulation. For instance, Gonadotropin-releasing hormone (GnRH) analogs can lead to pro-inflammatory changes in T lymphocytes [76]. TRP channels regulate fundamental biological processes throughout the body, and the dysfunction of these channels has been causally linked to a number of disease states [77] that regulate the innate immune cell function in lung inflammation [78]. The airway wall is an essential controller of inflammatory, immune and regenerative responses to viruses, and Asthma is a T lymphocyte-controlled disease of the airway wall caused by inflammation, thus a strong link between Asthma and infection with coronaviruses is established [79, 80]. Based on the GO and KEGG results, as mentioned above, it could be postulated that *A. annua* acts on COVID-19 mainly by regulating inflammatory responses, transcription, and proliferation.

Antimalarial drugs, for which lung concentration data are available, are found to be 10 to 160-fold more concentrated in the lungs than in blood [56]. Artemisinin-type compound possesses an antiviral ability [81]. Through the clinical observation, we primarily speculate that *A. annua* related drugs such as artemisinin piperaquine have considerable command on controlling the processes leading to lung inflammation.

The use of *A. annua* for COVID-19 could be mainly linked to its anti-inflammatory and immune regulatory properties. Its multiple active compounds aim for multiple targets, which were mapped to different pathways embodied in the complex integrated network mechanisms of multi-component, multi-targeted, and multi-channel regulated treatment of COVID-19. Although our study provides theoretical support and scientific evidence for *A. annua*’s capabilities in fighting against COVID-19, it has some limitations in terms of lacking specific experimental verification. Thus, our study puts foundation for further screening of these compounds and verification of the observations made with animal or cell experiments to clarify the main regulatory targets of *A. annua*.

In summary, in our study, we have used a network pharmacology strategy to predict the main active compounds and key targets of *A. annua* for the treatment of COVID-19 and speculated the potential mechanisms from multiple approaches and perspectives.

## Conclusion

Our study initially found *A. annua* to have anti-inflammatory and immune regulatory properties. Further, we analyzed the potential active compounds and targets of *A. annua* for COVID-19 using the network pharmacology methods. The screened results show that *A. annua* can prevent and treat COVID-19 through multiple components, targets, and pathways that need further validation through in vitro or in vivo studies.

## Data Availability

The datasets generated and analysed during the current study are available in the Zenodo repository (https://zenodo.org/record/5565195#.YWXPgvkzY2x).

## Abbreviations

*A. annua*: *Artemisia annua* L
ACTs: Artemisinin-based combination therapies
OB: Bioavailability
BP: Biological process
CC: Cellular component
C-T: Compound-target
C-T-D: Compound-target-disease
COVID-19: Corona Virus Disease 2019
DL: Drug-likeness
GO: Gene Ontology
GnRH: Gonadotropin-releasing hormone
KEGG: Kyoto Encyclopedia of Genes and Genomes
MD: Molecular dynamics simulation studies
MF: Molecular function
PPI: Protein-protein interaction
RMSD: Root mean square deviation
SARS-CoV-2: Severe acute respiratory syndrome coronavirus 2
TCMSP: Traditional Chinese Medicines for Systems Pharmacology Database and Analysis Platform
TRP: Transient receptor potential
